# [^18^F]-Fludarabine for Hematological Malignancies

**DOI:** 10.3389/fmed.2019.00077

**Published:** 2019-04-17

**Authors:** Louisa Barré, Narinée Hovhannisyan, Caroline Bodet-Milin, Françoise Kraeber-Bodéré, Gandhi Damaj

**Affiliations:** ^1^LDM-TEP Group, UMR6030 Imagerie et Stratégies Thérapeutiques des Pathologies Cérébrales et Tumorales, Caen, France; ^2^Service de Médecine Nucléaire, Centre Hospitalier Universitaire de Nantes, Nantes, France; ^3^Department of Hematology, University Hospital Center of Caen, Caen, France

**Keywords:** ^18^F-fludarabine, lymphoma, PET—positron emission tomography, imaging, diagnosis

## Abstract

With the emergence of PET/CT using ^18^F-FDG, molecular imaging has become the reference for lymphoma lesion detection, tumor staging, and response assessment. According to the response in some lymphoma subtypes it has also been utilized for prognostication of disease. Although ^18^F-FDG has proved useful in the management of patients with lymphoma, the specificity of ^18^F-FDG uptake has been critically questioned, and is not without flaws. Its dependence on glucose metabolism, which may indiscriminately increase in benign conditions, can affect the ^18^F-FDG uptake in tumors and may explain the causes of false-positive imaging data. Considering these drawbacks, ^18^F-fludarabine, an adenine nucleoside analog, was developed as a novel PET imaging probe. An efficient and fully automated radiosynthesis has been implemented and, subsequently preclinical studies in xenograft murine models of hematological maligancies (follicular lymphoma, CNS lymphoma, multiple myeloma) were conducted with this novel PET probe in parallel with ^18^F-FDG. The results demonstrated several crucial points: tumor-specific targeting, weaker uptake in inflammatory processes, stronger correlation between quantitative values extracted from [^18^]F-fludarabine and histology when compared to ^18^F-FDG-PET, robustness during immunotherapy with rituximab, divergent responses between CNS lymphoma and glioblastoma (GBM). All these favorable findings permitted to establish a “first in man” study where 10 patients were enrolled. In DLBCL patients, increased uptake was observed in sites considered abnormal by CT and [^18^F]FDG; in two patients discrepancies were observed in comparison with ^18^F-FDG. In CLL patients, the uptake coincided with sites expected to be involved and displayed a significant uptake in hematopoietic bone marrow. No uptake was observed, whatever the disease group, in the cardiac muscle and brain. Moreover, its mean effective dose was below the effective dose reported for ^18^F-FDG. These preclinical and clinical findings revealed a marked specificity of ^18^F-fludarabine for lymphoma tissues. Furthermore, it might well be a robust tool for correctly quantifying the disease, in the presence of confounding inflammatory processes, thus avoiding false-positive results, and an innovative approach for imaging hematological malignancies.

## Introduction

Cancer diagnosis has significantly been improved over the past decades, due to novel imaging agents that enable earlier detection. The challenge of an imaging technique is to demonstrate with accuracy the morphology and functional status of a tumor tissue. Historically, the staging and restaging of lymphoma have been established using CT. The higher accuracy of PET/CT using ^18^F-FDG in baseline lymphoma staging compared with traditional anatomical imaging techniques such as CT or MRI has profoundly changed the management of patients. This investigation appears as the most efficient in the initial assessment and appreciation of the therapeutic response. In addition, this approach affords important information in terms of prognosis and can lead to an optimization of the therapeutic strategy. Although PET/CT is a non-invasive imaging technique, which constitutes one of its major advantages, the findings using ^18^F-FDG may be misinterpreted to differentiate uptake within a site of cancer from uptake in a site of inflammation or infection. In fact, false-positives occur because ^18^F-FDG is taken up in any process associated with increased glycolysis such as inflammation, infection, or granulomatous disease. On the other hand, it is to highlight that normal physiological uptake of ^18^F-FDG into the brain, heart, digestive tract will mask the lesion, and hence downgrade the disease falsely. A consensus exists to consider ^18^F-FDG-PET more valuable in Hodgkin's disease and early-stage aggressive non-Hodgkin's lymphoma (NHL) and less useful in indolent NHL which represent 40% of all non-Hodgkin lymphoma subtypes (1–3).

Based on the characteristics of ^18^F-FDG-PET, novel imaging probe must be developed to fulfill the need of a more specific radiopharmaceutical for a better tumor delineation and a more precise evaluation of the response to therapy. To improve the diagnostic accuracy, in particular in lymphoma with a fluctuating ^18^F-FDG avidity, ^18^F-fludarabine was introduced as a novel PET probe. Though, ^18^F-fludarabine appears to be an appealing tool in evaluation of suspicious finding on ^18^F-FDG PET both before or after treatment (4).

Our approach was based on the therapeutic activity of fludarabine, alone or in combination with other active drugs, in the clinical treatment of lymphoid malignancies and more particularly in the treatment of lymphoma that have a low proliferative index. Fludarabine is transported into the cells and phosphorylated intracellularly into its triphosphate form, by the deoxycytidine kinase, the principal active compound. One of the characteristics of this drug is its cellular accumulation, which is cell-cycle-independent (5). This nucleoside analog which has a fluorine atom is resistant to deamination resulting in a therapeutic activity. To elaborate a probe for PET imaging, the fluorine atom was replaced by a fluorine-18. The manufacturing process, which includes efficiency of radiolabeling, purification, and stability of the final product, automation, was subject to various quality control tests prior to ^18^F-fludarabine implementation for *in vivo* studies (6).

## Radiosynthesis of ^18^F-Fludarabine

The most reliable radiosynthesis of ^18^F-fludarabine involved a simple two-step procedure. The strategy reported for the radiolabeling was based on a nucleophilic substitution of a nitro group at the two-position on the purine ring to act as a leaving group. The protected nitro precursor, described as a 2-nitro-pentabenzoyl adenosine derivative, was involved in a classical fluorination reaction using K^18^F/K222 followed by an intermediate purification on a Sep-Pak silica.

To generate ^18^F-fludarabine, hydrolysis of benzoyl groups using a mixture of methanol/aqueous ammonia was applied before a final HPLC purification. The robustness of the described process (radiochemical yield 48 ± 3%, specific activity 310 ± 72 GBq/μmol, radiochemical purity up to 99%) allowed us to initiate several preclinical studies and a first-in-man clinical trial (6).

## Preclinical Studies

In preliminary studies biodistribution or pharmacokinetic properties, metabolism, and dosimetry were established on control animals which are important prerequisites, before testing ^18^F-fludarabine on animal models of hematological malignancies.

### ^18^F-Fludarabine in Control Animals

The accumulated activity of ^18^F-fludarabine, over 1 h period and after i.v. injection (5–12 MBq), was preferentially in the spleen and the kidneys which, respectively, confirmed the selectivity for lymphoid organ, and demonstrated its renal excretion (7). Moreover, our *in vivo* findings indicated no degradation of the probe 60 min post injection, which is an ideal characteristic for an imaging agent. To estimate the maximum dosage of ^18^F-fludarabine that could be safely administrated to patients, radiation dose was calculated in major organs; the results revealed that the urinary bladder wall, considered as a limiting organ, received the highest dose. Nevertheless, the effective dose obtained by extrapolation of animal data to humans, was consistent (7.3 mSv) with the previously reported values of ^18^F-FDG (3.8–10.7 mSv) (8).

### ^18^F-Fludarabine in a Xenograft Model of Human Follicular Lymphoma

**Figure 1:**
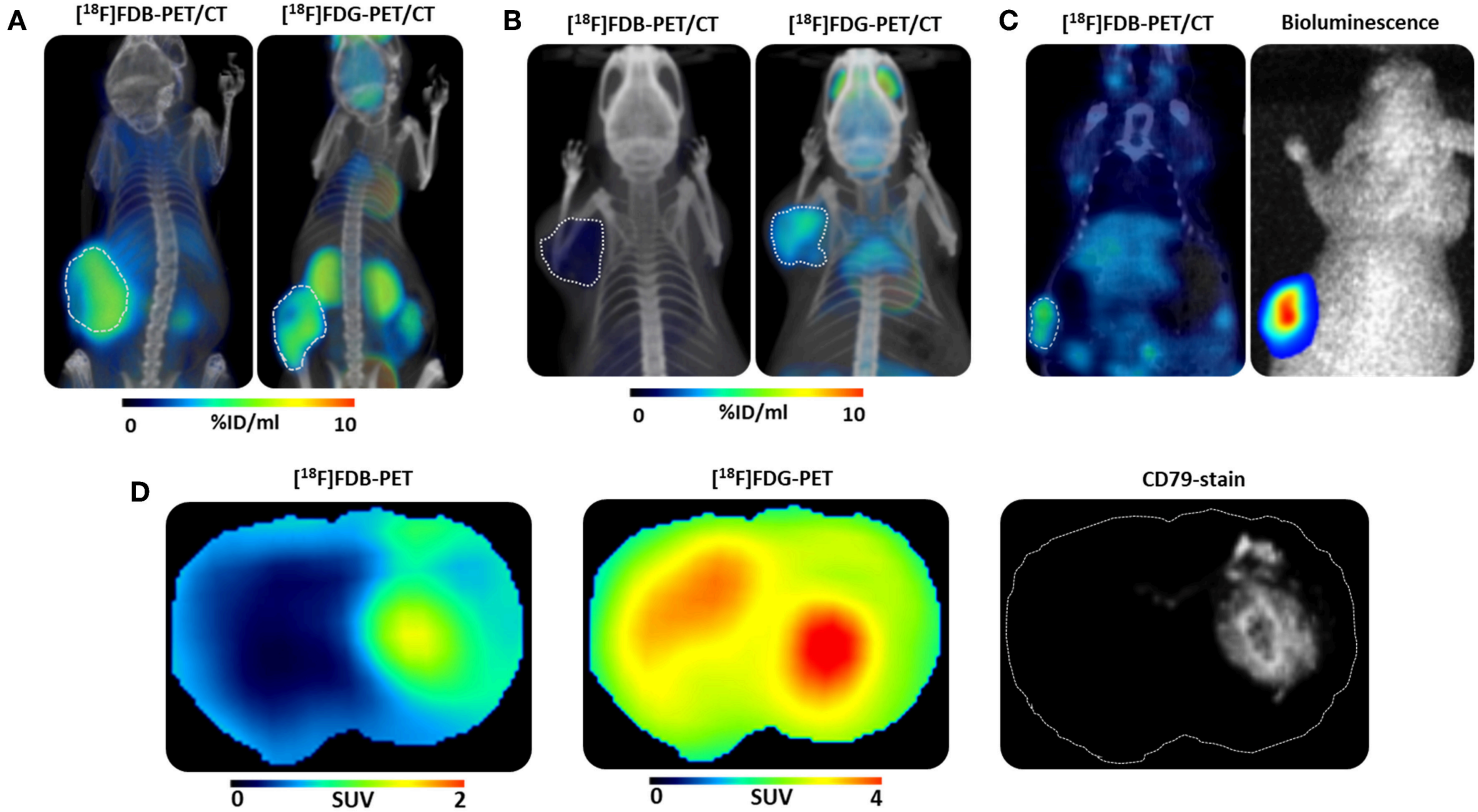
Preclinical studies. 3D illustration of μPET/CT fused scans with [^18^F]Fludarabine ([^18^F]FDB) and [^18^F]Fluorodeoxyglucose ([^18^F]FDG) in **(A)** mice bearing human follicular lymphoma xenograft, reproduced from Hovhannisyan et al. (10), no permission required **(B)** inflammation-bearing mouse, reproduced from Hovhannisyan et al. Copyright 2016 American Chemical Society (11), permission obtained **(C)** μPET/CT scan with [^18^F]FDB and corresponding bioluminescence image of a mouse bearing human multiple myeloma xenograft, reproduced from Hovhannisyan et al. (12), no permission required **(D)** Representative co-registered μPET scans and corresponding immunohistochemistry image of a mouse bearing human CNS lymphoma xenograft, reproduced from Hovhannisyan et al. (13), no permission required.

Follicular lymphoma is the most common subtype of indolent lymphoma. This lymphoma is FDG-avid and PET/CT using ^18^F-FDG is the current standard tool in its management in humans (9). To determine the potential of ^18^F-fludarabine and to acquire comparative preclinical data with ^18^F-FDG, biodistribution was carried out in parallel on a SCID xenografted tumor model. A marked difference in their behavior was observed which was in favor of ^18^F-fludarabine: in the tumor, its accumulation increased rapidly to reach a plateau within 20 min and its specific binding led to high-contrast images by comparison with ^18^F-FDG (Figure 1A). The clear positive correlation (*p* < 0.001) between the tracer uptake in the tumor and the density of lymphoid cells (determined by histological analysis) highlighted, the sensitivity of ^18^F-fludarabine. It was also important to demonstrate that the treatment with the anti-CD20 antibody rituximab did not have any negative influence on the tumor-targeting ability and, we hypothesized that ^18^F-fludarabine could be able to detect residual disease under treatment (10).

### ^18^F-Fludarabine in a Murine Model of Inflammation

The major drawback of ^18^F-FDG is its uptake in inflammatory tissue providing false-positives which could lead to a misinterpretation and perhaps an overtreatment of the patient. For this purpose, our reflex was to test ^18^F-fludarabine and compare to ^18^F-FDG in a previously described murine model of inflammation (14). The PET image analysis revealed that the uptake of this novel radiopharmaceutical in the inflamed tissue is negligible compared to ^18^F-FDG (Figure 1B). These results enhance the potential of ^18^F-fludarabine as a more specific probe (11).

### ^18^F-Fludarabine in Brain Tumors (CNS Lymphoma vs. Glioblastoma)

Primary central nervous system lymphoma (PCNSL) account for 5% of primary brain tumors and are predominantly diffuse large B cell lymphoma (90% of cases). MRI is the reference imaging for the diagnosis and monitoring (15). The evaluation of the therapeutic response, based on MRI, is perfectible. This imaging modality can miss atypical forms not enhanced by gadolinium and the significance of contrast enhancement occurring under or after treatment is sometimes ambiguous (16). ^18^F-FDG is established as the reference imaging in systemic lymphomas, but its applications in PSNCL are restricted by the limited specificity of cerebral fixations, and high uptake in healthy brain tissue (17). Taken into account the limitations of ^18^F-FDG, we demonstrated in a human CNS lymphoma model the pertinence to use ^18^F-fludarabine to detect brain lesions and we established its superiority over^18^F-FDG in differentiating brain tumors (13).

In the CNS lymphoma model, which closely mimics disseminated lesions, a marked retention was observed with ^18^F-fludarabine in accordance with histological findings (CD79 staining) representative of cells lymphoma (*p* < 0.001). ^18^F-Fludarabine exhibited tumor to background ratio (TBR) 2 to 3-fold higher than ^18^F-FDG, this made delineation of the tumor more precise. ^18^F-FDG, on the other hand, is poor in accurate delineation of the lesion due to its normal physiological brain uptake and poor specificity (Figure 1D). Considering high-grade glioma (GBM) and CNS lymphoma differentiation, the diagnostic accuracy is uncertain due to a similar imaging appearance on MRI or the previously described limitations of ^18^F-FDG. The scenario to use ^18^F-fludarabine is relevant taking into consideration that this probe has a rapid clearance from glioblastoma and this feature can help to discriminate between both brain tumors.

### ^18^F-Fludarabine in Multiple Myeloma (MM)

Multiple myeloma (MM) is a clonal plasma cells that accounts for 15% of all hematological malignancies. ^18^F-FDG is an accepted imaging technique to assess and monitor myeloma therapy. Despite the fact that ^18^F-FDG is reasonably sensitive and specific for bone disease, the detection of diffuse infiltration of plasma-cells in bone marrow, and lytic lesions in the skull is underestimated (1, 19). Based on our previous results in the animal models, ^18^F-fludarabine was then considered in a xenograft MM murine model. The tumor growth was followed by bioluminescence (BLI), after injection of a luciferase reporter MM cell line and characterized by immunohistochemistry (IHC, CD 138 staining) (Figure 1C). To compare with ^18^F-FDG, the metabolically active tumor was defined for both radiotracers (12). Although the ^18^F-FDG uptake was superior, the quantitative data extracted from IHC or BLI are in better agreement with the ^18^F-fludarabine uptake. These findings enforce the hypothesis that this radiopharmaceutical could be more suitable to detect MM disease.

## Clinical Study

The reported preclinical studies revealed the real potential of ^18^F-fludarabine to detect hematological malignancies and have resulted in the design of a clinical research protocol. This novel PET probe has been evaluated in human to better identify pathological from physiological or inflammatory uptake at initial staging of the disease and, in the future to enhance PET performance for therapeutic evaluation. Ten untreated patients with either B-cell chronic lymphocytic leukemia (B-CLL, *n* = 5) or diffuse large B-cell lymphoma (DLBCL, *n* = 5) were included in the study (18). CLL imaging with ^18^F-FDG-PET is not recommended, except in the case of suspected disease transformation (Richter syndrome), in contrast to DLBCL disease where it is being included as part of clinical practice. Nevertheless, despite an excellent sensitivity, the analysis of some areas remains difficult due to the lack of ^18^F-FDG specificity (bone marrow or spleen for example). Despite new criteria (20), the interpretation of ^18^F-FDG-PET positivity after therapy remains difficult, partly due to tumor-, and/or treatment-associated inflammation leading to false positives (4).

**Figure 2:**
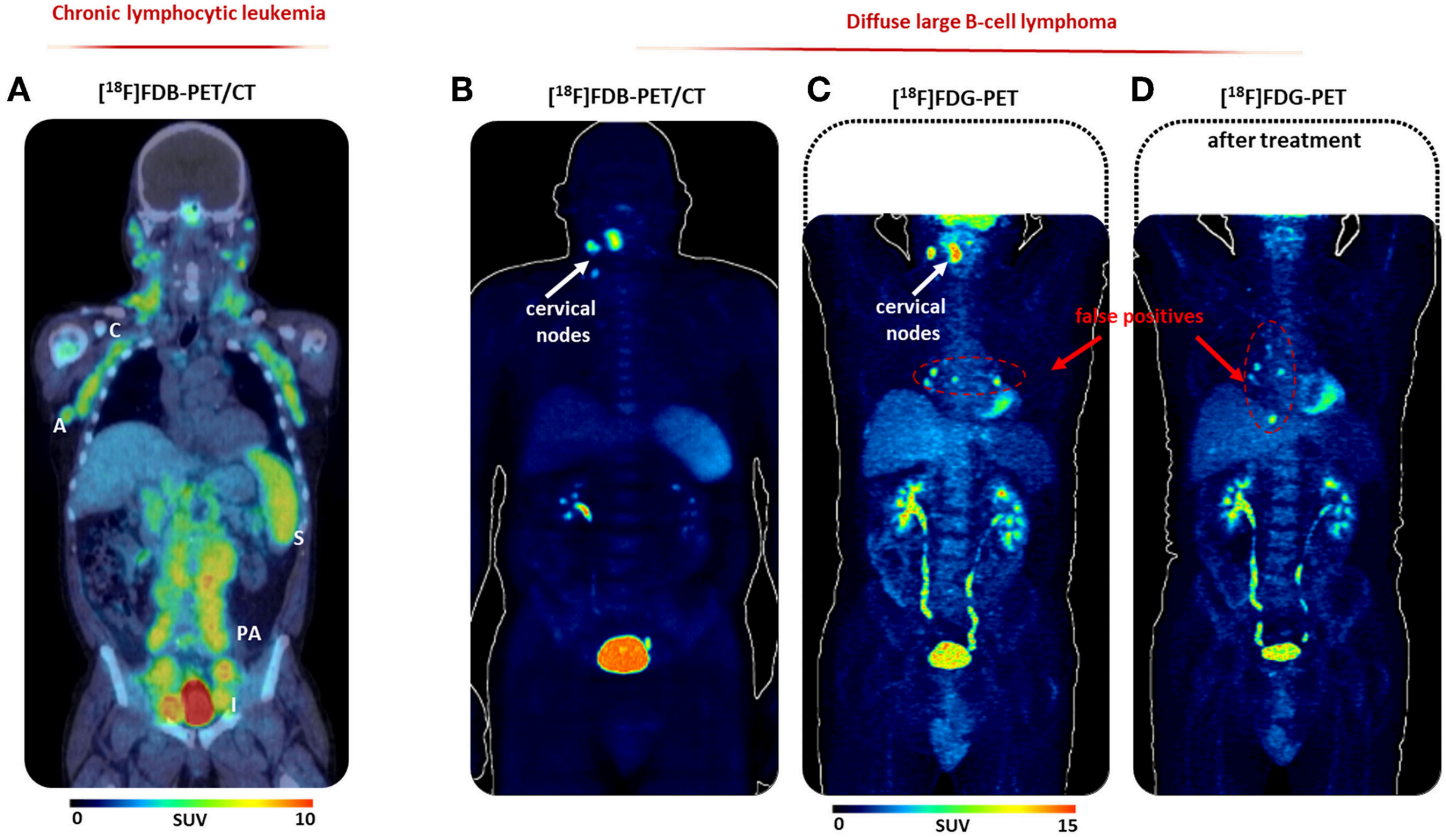
First-in-man study. **(A)** [^18^F]FDB-PET/CT (30–50 min scan period) of a representative CLL patient; **(B)** [^18^F]FDB-PET (30–50 min scan period), **(C)** [^18^F]FDG-PET (60–80 min), and **(D)** post-treatment (60–80 min) of a representative DLBCL patient, reproduced from Chantepie et al. (18), no permission required. A, axillary nodes; C, cervical nodes; I, iliac nodes; PA, paraaortic nodes; S, spleen.

The design of the pilot clinical trial was to acquire six successive partial body scans for 250 min (0–10, 15–25, 30–50, 90–100, 180–190, 240–250 min) after i.v. injection of ^18^F-fludarabine (4 MBq/kg) in both groups. In all patients, any side effects were observed to the radiopharmaceutical injection. The average activity received by the patients was 305 ± 76 MBq with a ^18^F-fludarabine mass of 0.23 ± 0.14 μg. The results with conventional modalities CT and [^18^F]FDG-PET (for DLBCL) were investigated. The imaging session was performed 60–80 min after injection of 335 ± 77MBq of ^18^F-FDG. In DLBCL patients, increased uptake of ^18^F-fludarabine was observed in sites deemed suspicious by CT and/or ^18^F-FDG. At 50 min, SUVs were significantly higher in involved lesions (SUVmax = 7.1) in comparison with histologically normal bone marrow (SUVmax = 2.3) or ascending aorta considered as reference (SUVmax = 1.4). In this group, aged 57–73 years, divergence was observed in two patients. In one patient a positivity with ^18^F-FDG was detected and not with ^18^F-fludarabine in bilateral hilar foci. These foci persisted at subsequent evaluation with ^18^F-FDG and were considered as false positives (Figures 2B–D). Indeed this patient was free from relapse more than 2 years after the end of treatment. In the second patient, unilateral testicular lymphomatous infiltration was not observed with ^18^F-fludarabine and could be attributed to the role of the testis barrier (21). In CLL patients, aged 51–70 years, ^18^F-fludarabine revealed all involved lymph nodes, with also a marked accumulation in the spleen and bone marrow involvement. At 50 min, SUVmax was 1.5 on the mediastinal vascular noise (taken as reference) against 6.05 in the affected lymph nodes, 7.7 for the spleen, and 4.4 in bone marrow, indicating a very good tumor/tissue contrast (Figure 2A). In both groups, no physiological uptake was noted in heart and brain.

## Conclusion

This recent study as a “proof of concept” in human paved the way to several underway national clinical trials including a larger cohort of patients to define the role and prognostic impact of ^18^F-fludarabine-PET/CT in the management of hematological malignancies. An exploratory, multicenter prospective clinical trial to evaluate the interest of PET images using ^18^F-fludarabine for initial staging and therapeutic evaluation in three subtypes of newly diagnosed lymphomas (DLBCL, Hodgkin lymphoma, and follicular lymphoma) is ongoing.

## Author Contributions

LB prepared a first draft of the manuscript. NH, CB-M, FK-B, and GD critically reviewed the manuscript. All authors conceived the idea of this review article and approved the final version.

### Conflict of Interest Statement

The authors declare that the research was conducted in the absence of any commercial or financial relationships that could be construed as a potential conflict of interest.
